# Phase contrast electron microscopy: development of thin-film phase plates and biological applications

**DOI:** 10.1098/rstb.2008.2268

**Published:** 2008-03-13

**Authors:** Kuniaki Nagayama, Radostin Danev

**Affiliations:** Okazaki Institute for Integrative Bioscience and National Institute for Physiological Sciences, National Institutes of Natural SciencesOkazaki 444-8787, Japan

**Keywords:** phase contrast, phase plate, electron microscope, transmission microscope, Zernike, Hilbert

## Abstract

Phase contrast transmission electron microscopy (TEM) based on thin-film phase plates has been developed and applied to biological systems. Currently, development is focused on two techniques that employ two different types of phase plates. The first technique uses a Zernike phase plate, which is made of a uniform amorphous carbon film that completely covers the aperture of an objective lens and can retard the phase of electron waves by *π*/2, except at the centre where a tiny hole is drilled. The other technique uses a Hilbert phase plate, which is made of an amorphous carbon film that is twice as thick as the Zernike phase plate, covers only half of the aperture and retards the electron wave phase by *π*. By combining the power of efficient phase contrast detection with the accurate preservation achieved by a cryotechnique such as vitrification, macromolecular complexes and supermolecular structures inside intact bacterial or eukaryotic cells may be visualized without staining. Phase contrast cryo-TEM has the potential to bridge the gap between cellular and molecular biology in terms of high-resolution visualization. Examples using proteins, viruses, cyanobacteria and somatic cells are provided.

## 1. Introduction

Biological transmission electron microscopy (TEM) has enabled visualization of many heretofore ‘invisible’ substances, including those that are soft, fragile or hydrous. Several innovative techniques are used to make this possible. Examples of biological sample transformations (and the respective techniques involved) include the conversion of soft substances to hard (chemical fixation), hydrous to non-hydrous (dehydration), fragile to robust (resin embedding) and the invisible to the visible (staining). These techniques have enriched the realm of microbiology and biological TEM, but are usually highly time-consuming, and thus the return is currently considered small compared with other methods. For instance, numerous innovations in the field of light microscopy, particularly the combination of fluorescent microscopy and metabolic tagging with fluorescent proteins, have been attracting significant interest as they permit visualization of molecular processes in living systems *in vivo*.

The decreased popularity of TEM studies in biology has resulted in the use of several new approaches. To resolve the issue of harsh sample treatment, vitrification (or ice embedding) with cryofixation has been introduced, where fragility, fluidity and softness of samples are all settled by the single decisive process of quick freezing (Taylor & Glaeser 1976; Heuser *et al*. 1979; Doubochet *et al*. 1982; Heuser 1983). To avoid electron beam damage to samples prepared with cryofixation, cryomicroscopy has been introduced to recover structural information with satisfactory results (Heide 1982; Fujiyoshi *et al.* 1991). These cryotechnologies have also been combined with tomography to give rise to electron cryotomography (Böhm *et al*. 2000), permitting three-dimensional reconstruction. Although each of the above techniques has shown some success, a fundamental issue remains to be solved: the high degree of transparency of cryofixed biological specimens to the high-energy electron beam.

To increase the visibility of transparent samples, a phase contrast method can be adopted. For instance, defocus phase contrast (DPC) has been an indispensable tool to study inorganic materials in their intact state (Reimer 1997). However, a fundamental problem with the application of this method to biological specimens is the resolution deterioration by defocusing. The solution to this problem in light microscopy suggests a similar approach here: a phase contrast method that uses phase plates. The present paper relates our experiences with this technique over the past decade.

Section 2 describes the phase contrast method using thin-film phase plates with an emphasis upon the practical aspects in experiments. Section 3 describes the synergistic combination of cryomicroscopy and the phase contrast method, namely phase contrast cryo-TEM. Section 4 describes the results of Zernike phase contrast (ZPC) cryo-TEM applied to proteins and viruses. The drastically improved phase contrast provided by phase plates is also described for Hilbert differential contrast (HDC) cryo-TEM in §5, where high-contrast images of bacterial and somatic cells in their vitrified state are reported. Finally, discussions and conclusions are given in §6.

## 2. Methods of phase contrast imaging

As transmitted waves (either light or electrons) pass through a sample under a microscope, the waves may be absorbed (changing their amplitude) and/or refracted (changing their phase). Samples that absorb transmitted waves to a high degree produce microscopic images primarily through alteration in wave amplitude. With transparent samples (called ‘phase objects’ in TEM), amplitude contrast does not occur and contrast arises instead through phase alteration. In light microscopy, the first phase visualization technique appeared in the Schlieren optics at the end of the nineteenth century, followed by the appearance of ZPC (Zernike 1942) and the Smith/Nomarski differential interference contrast (DIC; Smith 1947; Nomarski 1952) methods. DPC was originally developed by Scherzer for use in electron microscopy (Scherzer 1949). There have been many attempts to use phase plates that resemble those developed by Zernike in the context of TEM (Faget *et al*. 1962; Badde & Reimer 1970; Unwin 1970; Parsons & Johnson 1972; Johnson & Parsons 1973; Krawkow & Siegel 1975; Willasch 1975; Balossier & Bonnet 1981). All of these demonstrated some improvement in image contrast; however, various difficulties associated with manufacturing and charging of the phase plates prevented the techniques from being widely adopted. Recent research conducted by a Japanese group that devoted considerable time to phase plate development may solve most of the difficulties and make the use of phase plates practical (Nagayama 2005).

### (a) Defocus phase contrast TEM

Figure 1*a* demonstrates the process employed in conventional TEM. A circular aperture at the back focal plane (BFP) of the objective lens limits electrons contributing to image formation by intercepting those scattered outside the radius of the aperture. Two types of contrast generation are traditionally employed in the observation of biological samples by conventional TEM. If the sample is stained using heavy elements, some of the incident electrons are scattered at high angles and thus intercepted by the objective aperture; this produces amplitude contrast in the final image. As mentioned, however, procedures associated with this approach are problematic. This technique increases contrast but decreases resolution by introducing various artefacts in structural details. The aforementioned cryopreparation without staining is one way to alleviate this issue, but observation must rely on phase contrast.

In conventional TEM, phase contrast may be initiated by defocusing the objective lens (refer to figure 1*a*). Depending on the degree of defocus, the spectral characteristics of phase information transfer change. Figure 1*b* shows a plot of the phase contrast transfer function (CTF). The CTF describes the amount of optical information transfer as a function of the spatial frequency. It oscillates with an initial value of zero at the origin of the frequency or the centre of the diffraction space (*k*-space), thus acting as a band-pass filter. Low-frequency components are highly suppressed, leading to overall low contrast in images of unstained biological samples. Increasing the defocus value can improve the low-frequency transfer but greatly reduces the upper frequency bound and hence the resolution.

In practice, the defocus value is selected depending on the sample and the purpose of the observation. If the sample has characteristic size or periodicity, the defocus is selected so that the corresponding spatial frequency is in the middle of the first wave in the CTF oscillation. A so-called ‘defocus series’ is also often employed to cover a wider range of spatial frequencies. Usually, three or more images are taken at different defocus values and then combined.

### (b) Zernike phase contrast TEM

Figure 1*c* outlines the design of a ZPC TEM (ZPC-TEM). The only difference between ZPC-TEM and conventional TEM is the presence of a phase plate at the BFP, where the aperture supports the thin film of the plate. Mathematically, the BFP corresponds to a two-dimensional Fourier space characterizing the diffraction or the spatial frequency. Thus, the manipulation of frequency components at the BFP by the phase plates is equivalent to spatial filtering that is in turn able to manage phase contrast. The contrast transfer theory for ZPC-TEM, however, is beyond the scope of this paper and is developed elsewhere (Nagayama 1999; Danev & Nagayama 2001*a*,*b*). The CTF describing the characteristics of ZPC-TEM is shown in figure 1*d*. Uniform intact information transfer beginning at *k*=0 is apparent. The region of optimal information transfer is limited by the first zero-crossing in the CTF curve at the upper side. The low-frequency limit is determined by the size of the hole in the centre of the Zernike phase plate.

Extending information transfer towards the lower frequencies requires a reduction in hole size. Although there are some practical limitations, manufacturing phase plates with holes in the 100 nm range is not unfeasible using current technology, for instance using a focused ion beam apparatus. The precision of the mechanical alignment and stability of the phase plate should also be considered. Recently, heating of the phase plate was found to prevent beam-induced contamination (Hosokawa *et al*. 2005). In such a set-up, inserting and removing the phase plate in the beam path changes the thermal environment of the phase plate holder. This leads to a change in temperature and consequently to the thermal drift of the phase plate.

Specimen charging is one crucial factor that limits a straightforward handling of the phase plate. The beam tilt induced by specimen charge causes a shift of the optical axis at the diffraction plane (BFP). The hole in the phase plate must be large enough to accommodate these fluctuations of the optical centre.

### (c) Hilbert differential contrast TEM

The semicircular phase plate illustrated schematically in figure 1*e* is a recent invention (Danev *et al*. 2002). It can reveal the topographical features of images, similar to the Smith/Nomarski DIC. The phase contrast technique using this type of phase plate is called HDC TEM (HDC-TEM; Danev & Nagayama 2004), because the contrast it produces is based on the Hilbert transform (Lowenthal & Belvaux 1967).

The thickness of the phase plate film is adjusted to *π* phase shift. The electron beam corresponding to zeroth-order diffraction (namely *k*=0) is positioned at the centre of the supporting aperture, very close to but not touching the edge of the film. The contrast transfer theory of the HDC-TEM was developed extensively elsewhere (Danev & Nagayama 2004). The main characteristic of this technique is the antisymmetric CTF as shown in figure 1*f*. The modulus of the HDC-TEM CTF is the same as the ZPC-TEM CTF, and therefore the information transfer should be identical between the two techniques. However, the image produced by HDC-TEM possesses topographic information due to the unidirectional antisymmetry of the CTF.

In practice, there are differences in the information transfer between the HDC and ZPC TEMs. These are due to differences in the geometry of the phase plates and the positioning of the zeroth-order (central) beam at the BFP. The low-frequency cut-off for HDC-TEM is determined by the distance between the zeroth-order (central) beam and the phase plate edge. It can be arbitrarily adjusted, limited only by the size or shape of the central beam. Moderate specimen charging leads to deformation or deflection of the central beam, preventing its positioning too close to the phase edge.

For large objects such as cells and organelles, HDC-TEM is usually the optimal choice. By positioning the beam very close to the phase plate edge, the phase contrast transfer can be extended to much lower frequencies, which results in higher contrast images than those obtained by CTEM and ZPC-TEM. The HDC-TEM image data are usually interpreted directly without the need for further data processing or numerical reconstruction.

One of the disadvantages of using phase plates is electron loss due to scattering by the plate. The reason that carbon films are used for phase plates is that heavy elements such as metals cause greater electron loss. Nonetheless, signal reduction due to electron loss is unavoidable. Calculations using an acceleration voltage of 300 kV yield an estimated 25 and 50% loss for ZPC-TEM and HDC-TEM, respectively. This discrepancy in favour of ZPC-TEM is a result of the doubled thickness of the phase plate used in HDC-TEM. With a phase retardation of *π*/2, plate thickness is approximately 30 nm, while that for a phase retardation of *π* is approximately 60 nm at the acceleration voltage of 300 kV.

## 3. Phase contrast cryo-TEM

Sample preparation has always been one of the most challenging and crucial issues in applying TEM to biological samples. Three properties of biological specimens contribute to the difficulty in their preparation, which are as follows: (i) they are made of aqueous media, which are inappropriate for vacuum conditions, (ii) they consist of light elements (H, C, N and O), which weakly diffract electron waves, and (iii) their internal structure is frequently very complicated. A significant amount of time and effort has been devoted to resolving these issues, and a standard method has now been established, as shown in figure 2*a*.

This method has been widely used, but it does have several drawbacks, which include its destructiveness to the sample, possible introduction of artefacts and the significant amount of time required. Nonetheless, these drawbacks are tolerated due to the remarkable results obtained, examples of which are shown in figure 2*b*,*c*. Cyanobacterial cells were prepared using the above method with (figure 2*b*) and without staining (figure 2*c*). Although fine structures are visible in the stained samples as shown in figure 2*b*, these may well be artefactual.

The problems inherent in the traditional approach to sample preparation have largely been solved by introducing cryotechniques. Rapidly frozen vitrified (ice-embedded) specimens are much more probable than traditionally prepared samples to reflect the intrinsic internal structures of biological samples without interference from artefacts (Fernández-Morán 1960; Van Harreveld & Crowell 1964; Heuser *et al*. 1979).

The cryotechnique can be even more valuable when combined with a novel contrast enhancement technique using phase plates, and as such may give a useful artefact-free method to biological electron microscopists. Figure 2*d*,*f* provides an example depicting the cryopreparation process and the resultant images taken with HDC-TEM, respectively (Kaneko *et al*. 2005). Given that freezing is less harmful than other methods such as dehydration, this method is much more likely to be depicting the cyanobacterial cell in its *in vivo* state than those involving other preservation techniques. The fact that HDC-TEM also provides images with high contrast allows the intact fine structures to be easily recognized, as shown in figure 2*f*.

A cyanobacterial image obtained using a cryo-TEM technique that does not employ phase plates, DPC-TEM, is shown in figure 2*e*, depicting the same field as that shown in figure 2*f*. A comparison of the four images (figure 2*b*,*c*,*e*,*f*) clearly demonstrates the superiority of an approach that combines phase plate TEM and the cryotechnique.

## 4. Applications of ZPC cryo-TEM

Owing to the finite size of the central hole as described in §2, there is a limit to the recoverable lower spatial frequencies in ZPC. For instance, using a 300 kV TEM system and a Zernike phase plate with a hole size of 0.5 μm in diameter, this is approximately 0.02 nm^−1^ (approx. 50 nm). Given this limitation, the most efficient application of the ZPC-TEM to biology is structural studies of proteins (figure 3) and viruses (figure 4), which normally have geometries below 100 nm.

The experiments described below were carried out on a JEOL JEM-3100FFC electron microscope equipped with a field emission gun of the Shottky type, a cryostage cooled down to 50 K, an in-column energy filter and operated at a 300 kV acceleration voltage with or without a Zernike phase plate. The phase plates were made of an amorphous carbon film, which were designed to be 32 nm thick. The objective lens parameters were a spherical aberration coefficient of 3.7 mm, a chromatic aberration coefficient of 4.0 mm and a focal length of 5.0 mm. All observations were performed with a nominal magnification of ×10 000 to ×40 000 and an electron dose of approximately 1000 e^−^ per nm^−2^=10 e^−^ Å^−2^ in zero-loss filtering mode. The energy window width was set at 10 eV. A special aperture holder with a heating function was used to support the phase plates. To avoid contamination, the phase plates were kept at approximately 150°C at all times. All images were recorded with a Gatan Mega Scan 795 2K×2K charge-coupled device camera.

The samples were collected by centrifugation and dropped on holey carbon grids, which are perforated by equally sized small holes of 1 μm in diameter (Quantifoil). The sample in the aqueous thin-film stage was first carefully blotted with filter paper to remove excess liquid, and then rapidly frozen in liquid ethane using a rapid freezing device (LEICA EM CPC). The grid with the vitrified samples was transferred to the specimen chamber of the TEM using a cryotransfer system.

Single-particle analysis was performed on ZPC-TEM images obtained for a standard protein sample, a chaperonin GroEL, whose three-dimensional structure has been examined extensively by several groups (Ranson *et al*. 2001; Ludtke *et al*. 2004; Saibil 2006). The microscope was designed to cool the specimen to approximately 4 K using liquid helium. Our initial trials with single-particle specimens revealed that observation at a higher temperature (approx. 50 K) provided images with better contrast. This is in agreement with recent investigations on the influence of specimen temperature on data quality (Comolli & Downing 2005; Iancu *et al*. 2006). All data presented in this report were collected by allowing the liquid helium to evaporate, and then waiting for the specimen to warm up to 50–60 K. The temperature then stabilized via cooling through the outer liquid nitrogen shield, and imaging could be carried out without noticeable specimen drift.

The advantage of ZPC is illustrated in figure 3, which compares the DPC and ZPC images of GroEL (Danev & Nagayama 2008). With the conventional approach, the most challenging aspect of single-particle analysis is the first step: selecting the images that correspond to protein molecules. This is due to the fact that individual protein molecules must be identified using noisy raw images, which usually have a very poor contrast, as shown in figure 3*a*. This laborious particle selection procedure has recently been automated by the combination of sophisticated software and a high-speed computer (Mio *et al*. 2007). Nevertheless, the handling of ZPC cryo-TEM images with higher contrast, as shown in figure 3*b*, becomes less prone to human bias.

Once a sufficient number of particles have been sampled and particle selection is completed, analysis becomes more computer based and straightforward. A result of the three-dimensional reconstruction for GroEL based on approximately 1500 particles that were picked directly from raw ZPC cryo-TEM images is shown in figure 3*c*. In this study, which compared the analysis both with and without a Zernike phase plate (Danev & Nagayama 2008), a quantification of the efficiency of analysis was attempted by assessing attainable resolution at different levels of sampling, as shown in figure 3*d*. Judging by the correlation between spatial resolution and the number of particles, the analysis with ZPC-TEM seems to be 30% more efficient than that with conventional cryo-TEM.

Viruses have always been a central object of study of biological TEM. Negative staining was the first technique to radically improve virus visibility, while the second one was cryotrapping (Doubochet *et al*. 1982). ZPC-TEM promises to be the third method able to significantly increase the visibility of viruses in their intact form. Figure 4 shows ZPC-TEM images for influenza viruses (Sugawara *et al*. 2005; Yamaguchi *et al*. 2007).

In figure 4, the contrast improvement is evident by comparing the images derived from the DPC and ZPC techniques (figure 4*a*,*b*, respectively). Variations in the overall structure of virions are evident in figure 4*b*. Envelopes made of a lipid bilayer are clearly visible under the aligned outer spikes (figure 4*c*), and the inner core (figure 4*d*) depicts a regularity in packing of the genome. Variations in envelopes from immature to mature are also well resolved, as shown in figure 4*e*,*f*.

The structural preservation evident in the images allows one to characterize the virus as spherical with a 100–120 nm diameter, with spikes of approximately 26.3 nm in length, and possessing a lipid bilayer, which is approximately 6.4 nm thick. The spikes are thought to consist of haemagglutinin and neuraminidase molecules.

## 5. Applications of HDC cryo-TEM

The most important biological application of HDC-TEM is the use of phase contrast recovery to image an entire cell in an ice-embedded state. This fact may surprise electron microscopists because cells, particularly whole cells, are thought to be sturdy objects which should be invisible without sectioning. The cause of the unexpectedly impressive TEM images has yet to be fully determined, but the results should encourage the TEM community to favourably reconsider the applicability of phase contrast TEM. All of the experiments reported in this section were conducted with a 300 kV TEM system, with further details explained below. The experimental conditions were the same as those described in §4, except for the specific phase plate used and the fact that the somatic cells (PtK2) were cultured directly on a TEM grid.

The first example of cell imaging with HDC-TEM uses cyanobacterial cells (Kaneko *et al*. 2006, 2005). As shown in figure 2*e*, the HDC-TEM image can display topographic features and achieves an effect similar to that of DIC in light microscopy (Danev *et al*. 2002). Figure 5*a* shows high-contrast HDC-TEM micrographs of an ice-embedded cyanobacterial (*Synechococcus* sp. strain PCC 7942) whole cell (Kaneko *et al*. 2007). The thylakoid membranes, carboxysomes and polyphosphate bodies can be identified, along with the smooth cell walls that surround them (figure 5*a*). Identification was confirmed by comparison with conventional TEM images of ultrathin sections of chemically fixed and resin-embedded cyanobacteria of the same strain (figure 5*b*). However, the prominent structure shown by an arrow in figure 5*a* and determined to be a polyphosphate body using an electron spectroscopic imaging (Kaneko *et al*. 2007) cannot be observed by the standard approach due to the difficulty in preserving these structures during specimen preparation; as a result, they commonly appear as empty holes in ultrathin sections (figure 5*b*, arrow). Polyphosphate bodies often either shrink considerably during chemical fixation and dehydration, leaving spaces around them, or may be destroyed completely by the ultrathin sectioning process. In the conventional TEM image of ultrathin-sectioned cyanobacterial cells, fibrous structures believed to be DNA (figure 5*b*, arrowhead) are consistently observed radiating from the holes where a polyphosphate body was probably situated.

In addition to permitting visualization of intact ultrastructures, HDC-TEM allows the identification of cell constituents in their *in vivo* states. As such, we attempted to visualize DNA by the incorporation of BrdU (bromodeoxyuridine) into newly synthesized DNA, a technique widely used to investigate DNA synthesis *in vivo* in cell biological research. However, instead of labelling BrdU using fluorescent probes, a routinely used procedure, we initially attempted to take advantage of the elevated electron density of Br and visualize it directly. Rapidly growing cyanobacterial cells were cultured for 24 hours in liquid media containing BrdU, and the cells were then collected, rinsed thoroughly and frozen rapidly in liquid ethane.

HDC-TEM images of the BrdU-treated cells exhibit electron-dense areas (figure 5*c*, arrowhead) other than polyphosphate bodies. Electron-dense strands that extend from the polyphosphate bodies are also visible in this area of the HDC image (figure 5*c*, arrows). An almost identical electron-dense area is observed when the sample is visualized without a Hilbert phase plate (figure 5*d*), an image in which ultrastructural details are lost. This may reflect localization of Br via its high-contrast TEM, as would be expected given the incorporation of heavy elements into the DNA molecules (Kaneko *et al*. 2007). The existence of Br associated with the polyphosphate bodies indicates that at least part of the newly synthesized DNA colocalizes with the bodies.

While HDC ensures high-contrast imaging for rapidly frozen vitrified samples without staining, it requires rather thin specimens and is thus limited in its biological application. Fortunately, HDC-TEM may be used effectively with biological specimens as thick as 1 μm. Many prokaryotic cells are thin enough to be examined by HDC cryo-TEM without sectioning. A prerequisite for studying intact eukaryotic or mammalian cells by HDC-TEM is their ability to remain relatively flat on an electron microscope grid. The rat kangaroo cell line PtK2 meets this criterion, and we therefore chose it for imaging with HDC cryo-TEM.

Figure 6*a* shows a micrograph of a PtK2 whole cell by HDC cryo-TEM. Various membranous organelles are apparent, as are long filaments extending downwards from the cell periphery. The filaments have a width of 25 nm and are several micrometres long, the approximate size of microtubules. The fact that the filaments were eliminated by treatment with nocodazole further confirms their identity as microtubules (data not shown). Figure 6*b* shows a closer examination of the purified microtubules using HDC-TEM. Individual protofilaments can be discerned in this image, as can shadows of the depolymerized tubulin monomers.

As noted above, figure 6*a* reveals several membranous structures, and we identified some of them as mitochondria based on the unique appearance of their inner membranes. Figure 6*c* shows the cytoplasmic structures of PtK2 cells at higher magnifications. The ease with which one is able to identify mitochondria and other organelles using HDC-TEM is remarkable, as this is difficult to do with phase contrast light microscopes unless one uses specific probes or dyes such as MitoTracker.

## 6. Discussions and conclusions

Biological specimens for study with TEM are phase objects in which electrons are not absorbed under normal conditions (including specimens, both stained and unstained). It is therefore natural to employ the phase contrast method, particularly when enhanced with phase plates.

The weak phase-object approximation, which is considered to be a requirement for successful phase contrast methods, is described in almost all textbooks that deal with optics (Born & Wolf 1999). The assumption holds that the observed specimen must be thin enough to avoid significantly changing the phase of the wave incident on the specimen. Thus, resin-embedded specimens have traditionally been prepared as thin as possible, say, 100 nm thick or thinner.

Unexpected and remarkable results using very thick samples such as bacterial or somatic whole cells, as shown in figures 5 and 6, have cast doubt on the traditional prerequisites for specimen condition. The high-contrast HDC-TEM images of whole cells must be interpreted using a new contrast theory that might place greater importance on the optical coherence of the incident electron wave, similar to that developed for electron holography.

One as-yet unresolved issue is electron loss due to the phase plate. This problem may be successfully addressed by using a new class of phase plates made of microelectrodes or thin magnetic wires, which can control spatially extended electrostatic or vector potentials instead of using thin-film phase plates. Research on non-loss phase plates has already begun in several laboratories, with reports to appear elsewhere.

The trend in recent years has been observation of biological samples in their *in vivo* state, which usually means observation of vitrified samples. Phase contrast is the only way to extract information from such samples. However, maximum electron dose, which determines the strength of microscopic contrast, is limited when using unstained vitrified samples due to increased electron damage. Improvement of the phase contrast transfer of the electron microscope therefore becomes a burning issue. The recent developments in the application of thin-film phase plates to TEM show promising results as reported in this paper. As more experiments are performed, more information gathered and more experience accumulated, these new techniques will gain further popularity and acknowledgement in the EM community.

## Figures and Tables

**Figure 1:**
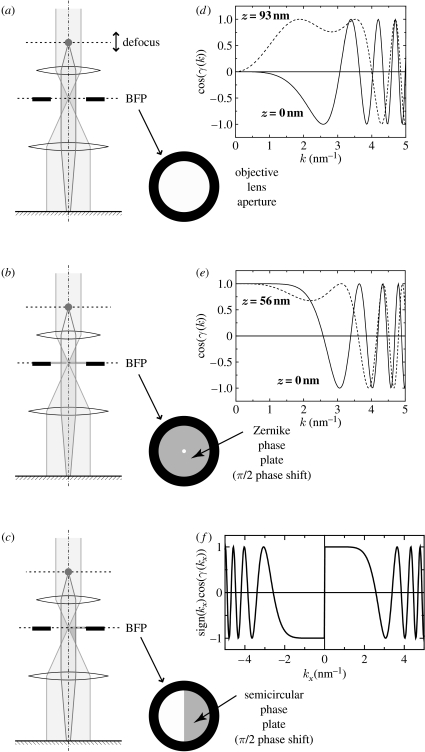
Three types of phase contrast methods. (*a*) Schematic illustrating the DPC with conventional TEM, in which contrast is adjusted by altering the defocus. (*b*) Contrast transfer function (CTFs) corresponding to DPC plotted against the modulus of the spatial frequency (*k*). (*c*) Schematic illustrating ZPC using a Zernike phase plate set at the back focal plane (BFP). (*d*) CTFs corresponding to ZPC plotted against the modulus of the spatial frequency (*k*) at the BFP. (*e*) Schematic illustrating the HDC using a Hilbert phase plate (semicircular phase plate) set at the BFP. (*f*) A CTF corresponding to HDC plotted against a unidirectional spatial frequency (*k*_*x*_). (*b*,*d*,*f*) 300 kV, *λ*=0.001968 nm and *C*_s_=3 mm. Adapted from fig. 1 of Danev & Nagayama (2006).

**Figure 2:**
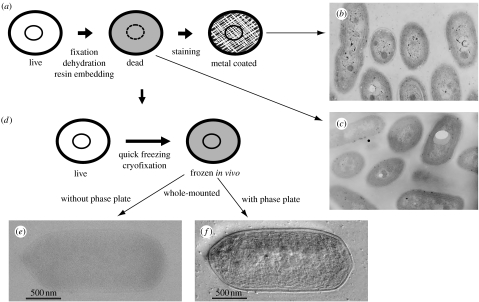
(*a*) A standard sample preparation and (*d*) an improved sample preparation using a cryotechnique. TEM micrographs associated with the preparation techniques are compared (*b*,*c*,*f*). (*b*) Conventional TEM images of a standard cyanobacterial cell sample (100 kV). (*c*) Conventional TEM images of a standard cyanobacterial cell sample without staining (100 kV). (*d*) A sample preparation using quick freezing. (*e*) A conventional TEM image for a vitrified cyanobacterial cell with deep defocusing (300 kV). (*f*) An HDC-TEM image for a vitrified cyanobacterial cell with a Hilbert phase plate (300 kV). Images (*e*,*f*) were adapted from fig. 1 of Kaneko *et al*. (2005).

**Figure 3:**
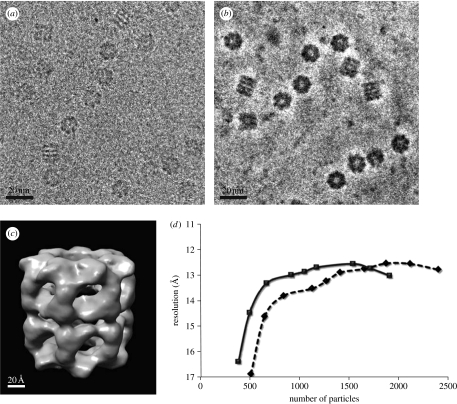
Single-particle analysis of a chaperonin GroEL based on ZPC-TEM. (*a*) A conventional TEM and (*b*) a ZPC-TEM micrograph of a vitrified GroEL sample obtained using a 300 kV TEM. (*c*) A three-dimensional reconstruction of GroEL with a nominal resolution of 1.23 nm. (*d*) Assessment of analysis efficiency by plotting the attained three-dimensional resolution against the number of particles sampled in the three-dimensional reconstruction (diamonds, CTEM; squares, ZPC-TEM). Adapted from figs. 3 and 5 of Danev & Nagayama (2008).

**Figure 4:**
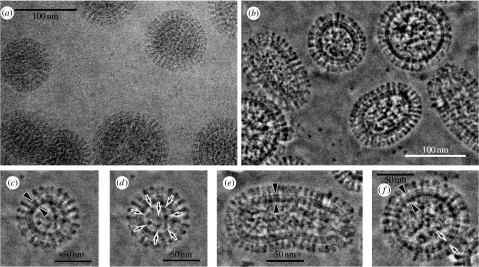
The 300 kV cryo-TEM images of vitrified influenza A viruses under (*c*) the plasma membrane and (*d*) an inner core. (*a*) A conventional TEM and (*b*) a ZPC-TEM micrograph for influenza A viruses. (*c*,*d*) Enlarged images of a spherical virion with a complete matrix layer (*c*, arrowheads) and an inner core (*d*, arrows). (*e*) An enlarged image of an elongated virion with a complete matrix layer (arrowheads). (*f*) An enlarged image of a spherical virion with a partial matrix layer (arrows). Adopted from figs. 1 and 2 of Yamaguchi *et al*. (2007).

**Figure 5:**
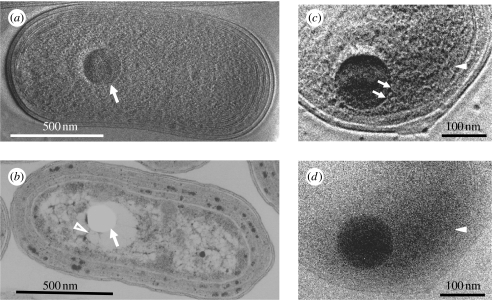
The 300 kV cryo-TEM images of vitrified cyanobacterial cells. (*a*) A 300 kV HDC-TEM image of a vitrified cyanobacterial whole cell. Polyphosphate body (arrow) is prominent among detailed ultrastructures. (*b*) A 100 kV conventional TEM image of a chemically fixed, resin-embedded and sectioned cell stained with uranyl acetate and lead citrate. Polyphosphate body is lost during the process and leaves an empty hole in the section (arrow). DNA fibres (arrowhead) extend from the hole. (*c*) An enlarged 300 kV HDC-TEM image of a cyanobacterial sample cultured in a BrdU-containing medium for 24 hours. (*d*) An enlarged 300 kV conventional TEM image using the same view field and experimental conditions are the same as given in (*c*), except with the use of a Hilbert phase plate. Adopted from figs. 1 and 2 of Kaneko *et al*. (2007).

**Figure 6:**
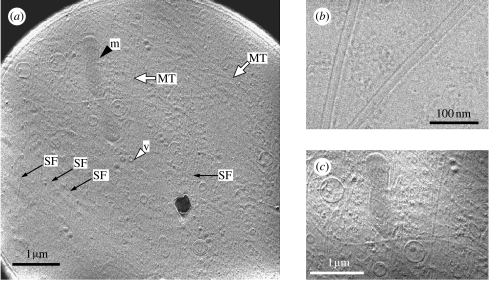
The 300 kV HDC-TEM images of a vitrified PtK2 whole cell. (*a*) An overall view of a PtK2 whole cell including microtubules (MT, white arrows), actin stress fibres (SF, black arrows) and different types of attached membranous organelles such as mitochondria (m, black arrowhead) and vesicles (v, white arrowhead). (*b*) An HDC-TEM image of vitrified microtubules purified *in vitro*. (*c*) A higher magnified view of (*a*) which is digitally contrast enhanced. Adopted from figs. 2, 5 and 6 of Setou *et al*. (2006).
